# Oropharyngeal administration of colostrum for preventing necrotizing enterocolitis and late-onset sepsis in preterm infants with gestational age ≤ 32 weeks: a pilot single-center randomized controlled trial

**DOI:** 10.1186/s13006-021-00408-x

**Published:** 2021-08-21

**Authors:** Xia OuYang, Chang-Yi Yang, Wen-Long Xiu, Yan-Hua Hu, Su-Su Mei, Qin Lin

**Affiliations:** Department of Neonatology, Fujian Provincial Maternity and Children’s Hospital, Affiliated Hospital of Fujian Medical University, Fuzhou, 350001 China

**Keywords:** Administration, oral, colostrum, premature birth, Enterocolitis, necrotizing, neonatal sepsis, Randomized controlled trial

## Abstract

**Background:**

Oropharyngeal administration of colostrum (OAC) may provide immunoprotective and anti-inflammatory effects that potentially reduce the incidence of necrotizing enterocolitis (NEC) and late-onset sepsis and improve short-term outcomes. Our objective was to evaluate the role of OAC in the early prevention of NEC and late-onset sepsis in preterm infants with gestational age (GA) ≤ 32 weeks.

**Methods:**

A pilot, single-center, 1:1 parallel randomized controlled trial was conducted in a 40-bed tertiary neonatal intensive care unit (NICU) in China from 1 January 2019 to 30 September 2020. Preterm infants were randomly divided into two groups with GA ≤ 32 weeks. The OAC group included preterm infants who received 0.4 ml of maternal colostrum via the oropharyngeal route every 3 h for 10 days beginning within the first 48 h after birth, and the control group included preterm infants who received normal saline instead. Data from the two groups were collected and compared.

**Results:**

A total of 127 infants in the OAC group and 125 infants in the control group were enrolled. The incidence of NEC (Bell stage 2 or 3) and late-onset sepsis were lower in the OAC group [2.36% vs. 10.40%, relative risk (RR) 0.23 (95% confidence interval (CI) 0.07, 0.78), adjusted RR 0.23 (95% CI 0.06, 0.84); 4.72% vs. 13.60%, RR 0.35 (95% CI 0.14, 0.85), adjusted RR 0.36 (95% CI 0.14, 0.95)]. In addition, the incidence of proven sepsis and intraventricular hemorrhage (IVH) (stage 3 or 4) were lower in the OAC group [2.36% vs. 8.80%, RR 0.27 (95% CI 0.08, 0.94); 1.57% vs. 7.20%, RR 0.22 (95% CI 0.05, 0.99)], and the time to achieve full enteral feeding was shorter (23.13 ± 9.45 days vs. 28.50 ± 14.80 days). No adverse reactions were observed in either group.

**Conclusions:**

Oropharyngeal administration of colostrum is a safe and simple NICU procedure that may yield a potential effect in decreasing the incidences of NEC, late-onset sepsis, and severe IVH and shorten the time to achieve full enteral feeding in preterm infants with GA ≤ 32 weeks.

**Trial registration:**

Chinese Clinical Trial Registry, ChiCTR1900023697, Registered 8 June 2019, retrospectively registered.

## Background

Necrotizing enterocolitis (NEC) and late-onset sepsis are major risk factors for mortality and morbidity in premature infants [1]. The incidence of NEC is 2–7% among infants with gestational age (GA) < 32 weeks and 5–22% among infants with birthweight (BW) < 1000 g; the rate of late-onset sepsis is 21% among infants with a GA of 25–28 weeks and 28% among infants with a BW of 751–1000 g [2]. Considerable evidence supports multifactorial mechanisms for NEC and late-onset sepsis. These mechanisms require the concurrent presence of an immature immune system (increased susceptibility) and triggers that lead to dysbiosis (disruption of the normal intestinal bacterial microbiome, resulting in increased growth of potentially pathogenic bacteria) as well as an exaggerated inflammatory host response with the release of cytokines and chemokines [3–6].

The oropharynx and intestinal tract of a fetus are continually exposed to immunoactive factors in amniotic fluid until 40 weeks of gestation is completed, and these factors stimulate the fetal immune system and accelerate intestinal maturation. Upon birth, breast milk replaces these functions of the amniotic fluid to a large extent. Breast milk is considered the perfect nutrition and contains a diverse array of microbiota and myriad biologically active components similar to amniotic fluid [7], including hepatocyte growth factor (HGF), transforming growth factor-β (TGF-β), immunoglobulin A (IgA) [8], platelet activating factor (PAF), lactoferrin and oligosaccharides etc. [9] In addition to being absorbed by the intestinal tract, these immunoactive factors can also produce specific benefits through oropharyngeal contact by “per oral breast milk feed”, which may promote immunocompetence by mechanisms of immunomodulation of cells within the oropharyngeal-associated lymphoid tissue (OFALT) and gut-associated lymphoid tissue (GALT) systems, and the mucosal absorption of factors that interfere with bacterial colonization. However, due to their incapability to receive “per oral breast milk feed” or clinical instability, most preterm infants with GA ≤ 32 weeks must be fed through a nasogastric tube (breast milk cannot pass through the oropharynx) in the early days after birth [10], and this treatment contributes substantially to NEC and sepsis risks due to the absence of oropharyngeal exposure to immunoactive factors during this critical period.

Therefore, some scholars have proposed using oral immune therapy to regulate the immune function of preterm infants and improve outcomes [11]. In 2009, Rodriguez first proposed the concept of “oropharyngeal administration of colostrum (OAC)” for very low birthweight (VLBW) premature infants, and the author suggested that OAC was a method to keep newborns exposed constantly to growth factors and protective biological factors just as in amniotic fluid within the early period after delivery [12]. Previous reports have shown immunoprotective, anti-inflammatory and intestinal maturity effects from a mother’s colostrum when administered early to the oropharyngeal pouch [13, 14]. However, theoretical debate and inconsistent findings remain. Therefore, this investigation aimed to explore whether OAC could safely reduce the incidences of NEC and late-onset sepsis in preterm infants with GA ≤ 32 weeks and improve their short-term prognosis.

## Methods

### Study setting

This study was a prospective, pilot, single-center, 1:1 parallel randomized controlled trial (RCT) conducted among preterm infants who were admitted to and cared for in the Department of Neonatology, Fujian Provincial Maternity and Children’s Hospital, a 40-bed tertiary neonatal intensive care unit (NICU), within 24 h after birth. This study was reviewed by the Ethics Committee of Fujian Provincial Maternity and Children’s Hospital (No: 2017–502) and was registered in the Chinese Clinical Trial Registry (Registration ID: ChiCTR 1,900,023,697).

### Participants

The inclusion criterion was a GA of less than 32 weeks. The exclusion criteria were as follows: (1) mothers prohibited from breastfeeding because of conditions including active tuberculosis or AIDS, treatment with radioisotopes (e.g., Iodine-131, Cobalt-60, Cesium-137), substance abuse or treatment with special medications excreted in breast milk, such as antimetabolic anticarcinoma agents (e.g., azathioprine, methotrexate, rituximab) or antipsychotics (e.g., lithium carbonate, clozapine); (2) birth complicated with severe gastrointestinal malformations (e.g., intestinal atresia, malrotation of intestine) or severe fatal disabling congenital chromosomal abnormalities (e.g., trisomy 21/18/13 syndrome); and (3) admission to the hospital with rapid serum antibody testing for cytomegalovirus IgG and IgM by enzyme-linked immunosorbent assay and urine cytomegalovirus DNA testing, indicating that the infant had a congenital cytomegalovirus infection. Informed consent was obtained from the guardians before they participated in the study.

For pregnant women diagnosed with inevitable premature delivery (potential recruits) during prenatal examinations in our hospital, our researchers communicated with them ahead of time and prescreened them before delivery. For pregnant women admitted to our hospital as an emergency or for those who did not undergo antenatal examinations at our hospital, our researchers immediately entered the ward for recruitment if the women gave birth to premature infants (GA ≤ 32 weeks). Informed consent was provided by the legal guardian (baby’s mother or father). The principal investigator (PI) and the other neonatologists participating in the trial actively worked in the neonatal and obstetric ward and had a schedule that guaranteed the constant presence (24 h per day) of the PI or at least one of the researchers responsible for recruiting participants, giving adequate information to parents, and following the ongoing study.

The hospitalization costs of the newborns were partly reimbursed by the Residents Basic Health Insurance (approximately 60%), but some needed to be paid by the parents themselves (approximately 40%). Parents were responsible for the costs of travelling to and from the hospital. The fathers had 14 days of paid paternity leave, and the mothers had 158 days. Our program did not provide additional subsidies for hospitalization costs of newborns, travel costs and working hours allowance of parents.

#### Study groups and experimental design

All the participants were separated into an OAC group and a control group using a random number table at admission by the appointed person. The nursing manager and statistician participated in the operation of ‘allocation concealment’ and did not involve in the recruitment of participants or the subsequent clinical work. First, the nursing manager compiled the allocation sequence table (the first column was the serial number of the recruited participant, the second column was the random number, and the third column was the group identification). Second, the nursing manager used the computer to generate a random number sequence, and the statistician assigned odd numbers to the intervention group and even numbers to the control group in advance. Then, each participant was assigned a unique computer-generated random number in sequence one by one. Finally, the three copies of allocation sequence Table (1 each for the nursing manager, PI and statistician) were sealed in an opaque envelope and locked away. Blinding for nurses who implemented the intervention was not possible given the nature of the intervention.

The detailed design of clinical experiment was as follows: (1) In the OAC group, breast milk was collected from both of the mother’s breasts with a hospital-grade electric milk pump within the first 24 h after birth at a frequency of 10 min on each side every 3 h (eight times per day). The breast milk was stored in clean, uniform, disposable, sealed cups marked with the infant’s name, admission number (AD), collection time and volume. The breast milk was sent from the families to the ward by cold-chain for nurses’ acceptance and registration into the Milk Bank Refrigerator, and this process was performed within 4 h after breast milk collection. OAC could be started once the breast milk was received. The primary nurse suctioned 0.4 ml of breast milk into a 1-ml syringe after the milk was warmed in a water bath and then dripped 0.2 ml of breast milk into each side of the infant’s oral cavity. With the help of a sterile silicone fingerstall, the breast milk was applied evenly on the cheeks, palate, lingual surface, gingiva and lips for no less than 2 min every 3 h (eight times per day). This treatment was started within 48 h after birth and lasted for a total of 10 days. (2) In the control group, the primary nurse used the same methods and frequencies of treatment as noted in the OAC group, but breast milk was replaced with normal saline. The respiratory rate, heart rate, and transcutaneous oxygen saturation of the infants were monitored during the procedure. The procedure was suspended when any of the following situations occurred: apnea, heart rate < 90 or > 180 beats/min, or transcutaneous oxygen saturation < 88%. The oral mucous membranes were observed after the procedure.

All the participants were observed until discharge. There was a specially assigned person to collect and register the clinical data. The date of discharge against medical advice or death was also recorded. The analyst was blinded to the allocation prior to analysis.

#### Routine care

With the exception of the different oral care interventions between the two groups, the feeding and treatment strategies were the same and performed according to the protocol of the NICU. It was advisable to increase the milk volume but not to exceed 20 ml/kg/d.

#### Quality improvement policy

On the day preterm infants (GA ≤ 32 weeks) were admitted to our NICU, a specially-assigned nurse provided promotional breastfeeding education to the parents, who were required to pass the training and demonstrate conformity before starting to collect and deliver breast milk. The clinician checked and recorded the status of breast milk delivery every day and provided feedback to the parents by telephone or WeChat (a mobile application offering an instant messaging service) with repeated emphasis on the importance of breast milk feeds to encourage them to continue to participate in the experiment, which was important for the OAC research to proceed smoothly in our NICU.

### Steering and monitoring of the trial

The Trial Steering Committee was established to oversee the advancement of the study, identify problems, and implement appropriate corrective measures. It was composed of the PI and two other researchers with sufficient clinical experience. Regular meetings were held by teleconference at least once per quarter throughout the trial.

An independent Data Monitoring Committee (DMC) was established to perform interim data analysis, investigate compliance with the trial, and monitor adverse events. The DMC included a biostatistician (the statistical analyst in this study) with rich experience in RCT research and two clinical professionals in neonatology who have extensive experience in premature infant research. The biostatistician conducted an interim analysis every 6 months, then evaluated the safety and effectiveness of OAC and decided whether to adjust the sample size based on the results of interim analysis.

The Clinical Trials Unit of Fujian Maternal and Child Health Hospital was involved to provide assistance in designing the research protocol and providing process supervision and data quality assurance.

The guideline for trial discontinuation was an incidence of serious adverse events likely related to the intervention of greater than 20%.

#### Baseline characteristics

The data collected in the standardized Access Database included the following: (1) the demographics and baseline characteristics of the infants, namely, sex, GA, birthweight, proportion of small for gestational age (SGA) infants, proportion of extremely low birthweight (ELBW) infants, proportion of sicker infants, head circumference at birth, 5-min Apgar score, Transport Risk Index of Physiologic Stability (TRIPS) score (including body temperature, respiratory status, systolic blood pressure, response to stimulation), diagnosis with neonatal respiratory distress syndrome (NRDS), use of pulmonary surfactant (PS), percentage of infants who had established enteral feeding within 24 h after birth, and time to start OAC/NS, and (2) the demographics of their mothers, including maternal age, pregnancy complications (diabetes, hypertension), premature rupture of membranes, confirmed chorioamnionitis by placental pathology, maternal medicine exposure (glucocorticoids, magnesium sulfate), number of fetuses and mode of delivery.

### Outcome assessment

1. The primary outcomes included the incidence of NEC (according to the modified Bell classification [15], Bell stage 2 or 3) and late-onset sepsis [16]. Late-onset sepsis, including proven and clinical sepsis, was defined as sepsis occurring at > 72 h of life. Proven sepsis was defined by a positive blood or cerebrospinal fluid culture that yielded a traditional neonatal pathogen, such as *E. coli*, *K. pneumoniae*, *P. aeruginosa*, etc., or a commensal species. Clinical or culture-negative sepsis was diagnosed by negative blood or cerebrospinal fluid culture and fulfillment of all the following criteria: a) 2 or more infection-related clinical manifestations; b) abnormal white blood cell count, C-reactive protein (CRP) level, platelet (PLT) count or procalcitonin (PCT) level; c) antibiotics used ≥5 days; and d) no evidence of concurrent focal infection, including pneumonia, urinary tract infection or necrotizing enterocolitis [17].
2. Secondary outcomes included the age of achieving full enteral feeding (160 ml/kg.d), full oral feeding, rate of weight gain (g/kg.d), days of mechanical ventilation and noninvasive ventilation, indwelling time of a peripherally inserted central catheter (PICC), breast milk feed rate (percent of infants whose total number of breast milk feed at discharge accounted for greater than 50% of the total number of intestinal feed), breast milk feed days (total number of days of breast milk feed during hospitalization including at least one meal of exclusive breast milk each day), and length of hospitalization. Related complications included patent ductus arteriosus [18] (PDA), severe intraventricular hemorrhage (IVH) [19] (diagnosis and classification by ultrasound as stage 3 or 4), bronchopulmonary dysplasia [20] (BPD, oxygen > 30% at 36 weeks corrected gestation), periventricular leukomalacia [21] (PVL), retinopathy of prematurity [22] (ROP) and cholestasis.
3. Apnea, bradycardia (defined as a heart rate < 90 beats/min), tachycardia (defined as a heart rate > 180 beats/min), desaturation (defined as a low saturation less than 88%) and oral mucous membrane injury were recorded as possible side effects.

### Sample size calculation

This study was a 1:1 parallel design RCT. We calculated the sample size based on the calculation formula for two independent proportions (pooled) [23]. The annual incidence of NEC without OAC among preterm infants (GA ≤ 32 weeks) in our NICU was approximately 10–12% according to medical records from the past 5 years. The incidence of NEC was estimated to be reduced by 50% in the OAC group (the incidence was estimated to be 5–6%) based on a literature review [24] and pre-experimental results, and the lost to follow-up rate was assumed to be 5%. Sample sizes of 374 participants in the OAC group and 374 participants in the control group were calculated using PASS 11.0. In-hospital data were analyzed among infants with GA ≤ 32 weeks admitted over the past decade, and the expected number of infants during the research period meeting the inclusion criteria and planned for enrollment was calculated to be 250 per year. Thus, the estimated period of our research was 3 years, and the target recruitment population was 750 participants. However, the sample size might be recalculated based on the results of the interim analysis.

$$ N=\frac{2{\left[{Z}_{\raisebox{1ex}{$a$}\!\left/ \!\raisebox{-1ex}{$2$}\right.}\sqrt{2\overline{\mathrm{p}\mathrm{q}}+}{\mathrm{Z}}_{\beta}\sqrt{{\mathrm{p}}_0{\mathrm{q}}_0+{\mathrm{p}}_1{\mathrm{q}}_1}\right]}^2}{{\left({\mathrm{p}}_0\hbox{-} {\mathrm{p}}_1\right)}^2}\left(\upalpha =0.05,\upbeta =0.1\right) $$

### Statistical analysis

Statistical analysis was performed using SPSS statistical software (SPSS for IBM. Version 23.0.0). Data were expressed in mean ± standard deviation, median (interquartile range), or number (percentage). Kolmogorov-Smirnov’s and Levene’s tests were used to assess the normality and homogeneity of variance for measurement data, respectively. Data were analyzed with student’s t-test for comparison of continuous parametric variables, the Mann-Whitney U test for continuous nonparametric variables, the χ2 test or Fisher’s exact test for categorical variables, and the multiple independent samples Kruskal-Wallis H test for one-way and orderly variables, respectively. Adjusted analyses for the main outcomes were conducted by Cox regression analysis and linear regression analysis. The relative risk was adjusted based on sex, birthweight (g), gestational age (weeks), TRIPS score, and histologic chorioamnionitis for the efficacy outcomes (NEC, LOS, IVH, age of achieving full enteral feeding) from the OAC intervention. A *P*-value of less than 0.05 was considered significant.

ELBW infants, sicker infants defined as those whose mechanical ventilation was started within the first three postnatal days and continued for at least 3 d or until death, and histologically diagnosed maternal chorioamnionitis-exposed infants defined as those whose mothers had confirmed chorioamnionitis by placental pathology were most at risk for NEC or late-onset sepsis and might not have tolerated the OAC intervention. Therefore, subgroup analyses were conducted in ELBW infants, sicker infants and histologically diagnosed maternal chorioamnionitis-exposed infants.

## Results

### The flow of participants through the stages of the trial is shown in Fig. 1

The planned period of our research was 3 years, and the target recruitment population was 750 participants. After receiving three interim analysis reports after one and a half years of the trial, the sample size was recalculated as 260 participants based on the decline in NEC occurrence (from 10.0 to 2.0%). We terminated the study after 21 months of work for several reasons (discussed in the Limitations section) and published this research as a pilot study. OAC is being implemented as a continuous quality improvement program in the nursing procedures of our hospital. The long-term outcomes are being followed-up, especially with regard to neurobehavioral development in the collection age of 3, 6, 12 and 18 months based on Bayley-III Scales of Infant development.

**Fig. 1:**
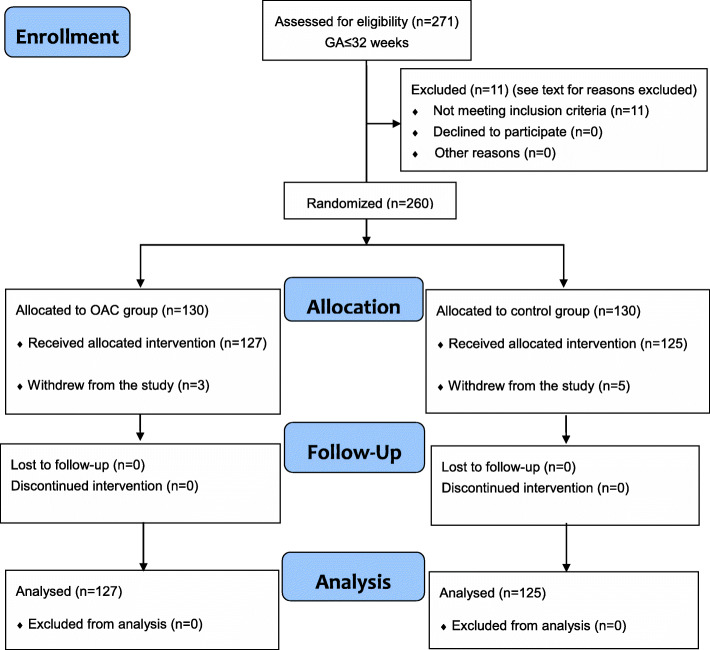
Participant flow diagram according to consolidated standards of reporting trials

A total of 271 premature infants met the inclusion criteria during the period of recruitment from 1 January 2019 to 30 September 2020 and 11 infants were excluded (including 5 due to contraindications for breast milk feeding in the mothers, 1 with Pierre-Robin syndrome, 1 with a congenital chromosomal abnormality, 1 with congenital intestinal atresia, and 3 with congenital cytomegalovirus infection). A total of 260 participants were included: 130 in the control group and 130 in the OAC group. Eight participants, including 5 in the control group and 3 in the OAC group, were withdrawn from the study because their guardians required discharge against medical advice due to family economic difficulties within 7 days of hospitalization.

The male-to-female sex ratio was 3:5, the mean GA was 27.00 ± 0.90 weeks, the birthweight was 932.38 ± 104.95 g, and the length of hospitalization was 2.62 ± 1.30 days. All of these participants died within 5 days after discharge (3 in the control group and 3 in the OAC group died within 24 h after discharge, and 2 infants in the control group died 3 and 5 days after discharge, respectively); none of these participants were diagnosed with NEC or late-onset sepsis. A total of 252 participants, including 125 participants in the control group and 127 participants in the OAC group, finally received the corresponding intervention, were followed up to discharge and were analyzed for the outcomes. All of these participants were discharged from the hospital after recovery, and no cases of death or loss to follow-up occurred.

### Maternal and neonatal general characteristics are shown in Table 1

There were no differences in the demographics or baseline characteristics. No cases of oral mucous membrane injury were observed in either group. For all the infants in this study, oxygen saturation measures remained stable or decreased slightly during the intervention. There were no episodes of apnea, bradycardia or tachycardia.

**Table 1:** Demographic and baseline characteristics in infants

Demographics	OAC group (*N* = 127)	Control group (*N* = 125)	*t/x*^*2*^*-*value	*df*	*P-*value	*Relative risk/Mean difference (95% CI)*
Sex (male), n (%)	65 (51.18)	78 (62.40)	3.23	1	0.07	0.82 (0.66, 1.02)
Gestational age (weeks), mean ± SD	30.00 ± 1.83	29.65 ± 2.04	1.45	250	0.15	2.48 (−0.89, 5.85)
Birthweight(g), mean ± SD	1, 302.26 ± 209.81	1, 328.72 ± 222.17	−0.97	250	0.33	− 26.46 (−80.07, 27.15)
Small for gestational age, n (%)	22 (17.32)	18 (14.40)	0.40	1	0.53	1.20 (0.68, 2.13)
Extremely low birthweight infant, n (%)	14 (11.02)	11 (8.80)	0.35	1	0.56	1.25 (0.59, 2.65)
Sicker infants, n (%)	20 (15.75)	22 (17.60)	0.16	1	0.69	0.90 (0.52, 1.56)
Birth head circumference (cm), mean ± SD	27.16 ± 1.50	27.08 ± 1.78	0.42	250	0.68	0.09 (−0.32, 0.50)
Apgar score at 5 min, median (IQR)	9 (9-10)	9 (9-10)	7, 646.00	-^a^	0.40	-^b^
TRIPS score, median (IQR)	13 (8-13)	13 (8-13)	8, 848.00	-^a^	0.06	-^b^
NRDS, n (%)	39 (30.71)	30 (24.00)	1.43	1	0.23	1.28 (0.85, 1.92)
Pulmonary surfactant use, n (%)	37 (29.13)	30 (24.00)	0.85	1	0.36	1.21 (0.80, 1.84)
Time to start OAC/NS (h), mean ± SD	24.47 ± 11.16	23.78 ± 10.92	0.50	250	0.62	0.70 (−2.04, 3.44)
Established enteral feeding within 24 h after birth, n (%)	111 (87.40)	105 (84.00)	0.60	1	0.44	1.04 (0.94, 1.15)
Maternal age (y), mean ± SD	31.81 ± 4.51	31.83 ± 4.79	−0.03	250	0.98	−0.02 (−1.17, 1.14)
Gestational hypertension, n (%)	23 (18.11)	15 (12.00)	1.84	1	0.18	1.51 (0.83, 2.76)
Gestational diabetes mellitus, n (%)	42 (33.07)	34 (27.20)	1.03	1	0.31	1.22 (0.83, 1.78)
Antenatal steroid use, n (%)	115 (90.55)	115 (92.00)	0.17	1	0.68	0.98 (0.91, 1.06)
Antenatal magnesium sulfate use, n (%)	82 (64.57)	68 (54.40)	2.70	1	0.10	1.19 (0.97, 1.46)
Twin or multiple gestation, n (%)	48 (37.80)	42 (33.60)	0.48	1	0.49	1.13 (0.81, 1.57)
Cesarean delivery, n (%)	77 (60.63)	71 (56.80)	0.38	1	0.54	1.07 (0.87, 1.31)
Premature rupture of membranes, n (%)	50 (39.37)	60 (48.00)	1.91	1	0.17	0.82 (0.62, 1.09)
Histologic chorioamnionitis, n (%)	42 (33.07)	33 (26.40)	1.34	1	0.25	1.25 (0.85, 1.84)

*TRIPS* transport risk index of physiologic stability, *NRDS* neonatal respiratory distress syndrome, *OAC* oropharyngeal administration of colostrum, *IQR* interquartile range, *SD* standard deviation. Sicker infants were defined as infants whose mechanical ventilation was started within the first 3 postnatal days and continued for at least 3 days or until death

^a^ Nonparametric test: df cannot be calculated

^b^ Nonparametric test: mean difference and its 95% CI cannot be calculated

### A comparison of the incidences of NEC and late-onset sepsis between the OAC and control groups is shown in Table 2

There were 13 cases of NEC (Bell stage 2 or 3) in the control group, including 4 cases of stage 2A, 6 of stage 2B, 2 of stage 3A, and 1 of stage 3B. All 3 infants with stage 3 NEC underwent surgery. There were 3 cases with NEC (Bell stage 2 or 3) in the OAC group, all of whom had stage 2A NEC and recovered without surgery. The incidence of NEC (Bell stage 2 or 3) was lower in the OAC group than in the control group [3/127 (2%) vs. 13/125 (10%), RR 0.23 (95% CI 0.07, 0.78), adjusted RR 0.23 (95% CI 0.06, 0.84)]. There were 17 cases of late-onset sepsis in the control group, including 6 cases of clinical sepsis and 11 cases of proven sepsis (3 cases of *Candida haemulonii*, 1 of *E. aerogenes*, 1 of *E. faecium* D group, 1 of *E. faecalis* D group, 1 of *E. coli*, 2 of *K. pneumoniae*, and 2 of *E. cloacae*). There were 6 cases of late-onset sepsis in the OAC group, including 3 cases of clinical sepsis and 3 cases of proven sepsis (1 case of *K. pneumoniae*, 1 of Staphylococcus bred, and 1 of *Candida haemulonii*). The incidences of late-onset sepsis and proven sepsis were lower in the OAC group than in the control group. However, there was no difference in the incidence of proven sepsis between groups as determined by adjusted analyses. [LOS: 6/127 (5%) vs. 17/125 (14%), RR 0.35 (95% CI 0.14, 0.85), adjusted RR 0.36 (95% CI 0.14, 0.95); proven LOS: 3/127 (2%) vs. 11/125 (9%), RR 0.27 (95% CI 0.08, 0.94), adjusted RR 0.31 (95% CI 0.08, 1.14)].

**Table 2:** Clinical outcomes between the OAC group and control group at the time of discharge

Clinical outcome	OAC group (*N* = 127)	Control group (*N* = 125)	*t/x*^*2*^-value	*P*-value	*Relative risk/Mean difference (95% CI)*	*Relative risk/ Mean difference* (95% CI)*	*Rate difference (95% CI)*
NEC (Bell stage 2 or 3), n (%)	3 (2.36)	13 (10.40)	6.85	**0.01**	0.23 (0.07, 0.78)	0.23 (0.06, 0.84)	8.04% (1.36, 15.31%)
Bell stage 2, n (%)	3 (2.36)	10 (8.00)	4.09	0.04	0.30 (0.08, 1.05)		5.64% (−0.62, 12.45%)
Bell stage 3, n (%)	0 (0.00)	3 (2.40)	-^a^	0.12	-^c^		2.40% (−1.67, 7.38%)
Late onset sepsis, n (%)	6 (4.72)	17 (13.60)	5.98	**0.01**	0.35 (0.14, 0.85)	0.36 (0.14, 0.95)	8.88% (1.12, 16.93%)
Clinical sepsis, n (%)	3 (2.36)	6 (4.80)	-^a^	0.33	0.49 (0.13, 1.93)		2.44% (−3.23, 8.49%)
Proven sepsis, n (%)	3 (2.36)	11 (8.80)	4.98	**0.03**	0.27 (0.08, 0.94)	0.31 (0.08, 1.14)	6.44% (0.04, 13.41%)
Patent ductus arteriosus, n (%)	45 (35.43)	33 (26.40)	2.41	0.12	1.34 (0.92, 1.95)		9.03% (−2.93, 20.64%)
Intraventricular hemorrhage (grade 3 or 4), n (%)	2 (1.57)	9 (7.20)	4.78	**0.03**	0.22 (0.05, 0.99)	0.32 (0.06, 1.65)	5.63% (−0.22, 12.17%)
Periventricular leukomalacia, n (%)	0 (0.00)	1 (0.80)	-^a^	0.50	-^c^		0.80% (−2.94, 5.03%)
Bronchopulmonary dysplasia, n (%)	7 (5.51)	10 (8.00)	0.62	0.43	0.69 (0.27, 1.75)		2.49% (−4.60, 9.76%)
Retinopathy of prematurity, n (%)	18 (14.17)	16 (12.80)	0.10	0.75	1.11 (0.59, 2.07)		1.37% (−7.78, 10.49%)
Cholestasis, n (%)	3 (2.36)	9 (7.20)	3.25	0.07	0.33 (0.09, 1.18)		4.84% (−1.28, 11.48%)
Age of achieving full enteral feeding (d), mean ± SD	23.13 ± 9.45	28.50 ± 14.80	−3.44 (df250)	**0.01**	−5.38 (−8.45, −2.30)	−4.54 (− 7.04, − 2.01)	-^b^
Age of achieving full oral feeding (d), mean ± SD	34.14 ± 16.27	35.03 ± 17.54	−0.42 (df250)	0.68	−0.89 (−5.09, 3.31)		-^b^
Rate of weight gain (kg.d), mean ± SD	13.47 ± 3.83	13.20 ± 3.42	0.58 (df250)	0.56	0.27 (−0.64, 1.17)		-^b^
Indwelling time of PICC (d), mean ± SD	24.67 ± 11.44	24.86 ± 13.19	−0.12 (df250)	0.91	−0.19 (−3.25, 2.88)		-^b^
Days of mechanical ventilation (d), mean ± SD	1.02 ± 3.02	1.94 ± 9.29	−1.06 (df250)	0.29	−0.92 (−2.63, 0.79)		-^b^
Days of noninvasive ventilation (d), mean ± SD	14.74 ± 14.31	13.49 ± 13.26	0.72 (df250)	0.47	1.25 (−2.17, 4.68)		-^b^
Breast milk feed rate, n (%)	108 (85.04)	103 (82.40)	0.32	0.57	1.03 (0.93, 1.15)		2.64% (−7.11, 12.39%)
Days of breast milk feeds (d), mean ± SD	30.41 ± 20.92	29.93 ± 17.97	0.20 (df250)	0.85	0.48 (−4.36, 5.32)		-^b^
Length of hospitalization (d), mean ± SD	41.12 ± 16.31	43.14 ± 17.15	0.96 (df250)	0.34	−2.02 (−6.17, 2.13)		-^b^

*IQR* interquartile range, *SD* standard deviation, *PICC* peripherally inserted central catheter

^a^ Fisher’s exact test: *χ*^2^ value cannot be calculated

^b^ measurement data: Rate difference and 95% CI cannot be calculated

^c^ RR and 95% CI cannot be calculated

*The relative risk/mean difference was adjusted for sex, birthweight (g), gestational age (weeks), TRIPS score, and histologic chorioamnionitis

### A comparison of the secondary outcomes between the OAC and control groups is shown in Table 2

**Fig. 2:**
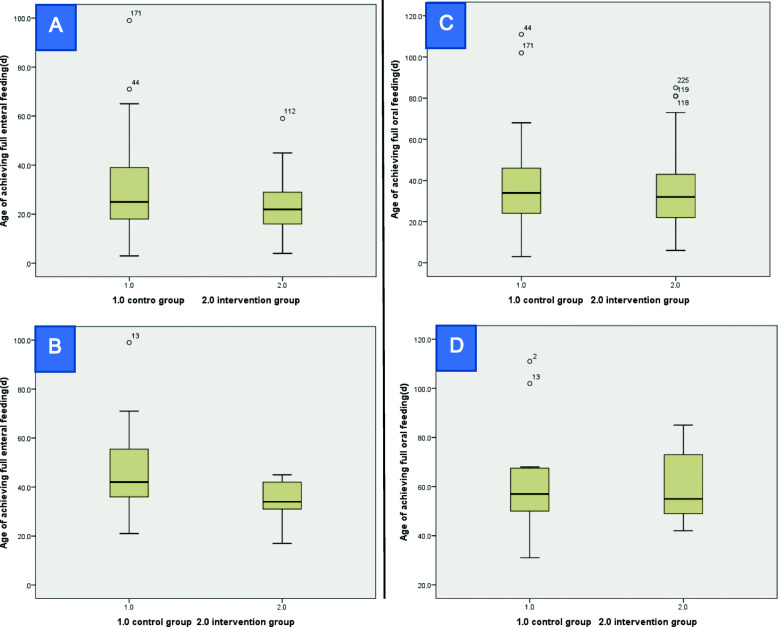
A comparison of the age of achieving full enteral/full oral feeding between the OAC and control groups among all infants/ELBW infants. **A** A comparison of the age of achieving full enteral feeding between the OAC and control groups among all infants. **B** A comparison of the age of achieving full enteral feeding between the OAC and control groups among ELBW infants. **C** A comparison of the age of achieving full oral feeding between the OAC and control groups among all infants. **D** A comparison of the age of achieving full oral feeding between the OAC and control groups among ELBW infants. This box plots showed the effects of OAC, which shortened the time to achieve full enteral feeding for all infants (**A**) and ELBW infants (**B**) but did not lower the age of achieving full oral feeding for all infants (**C**) or ELBW infants (**D**) in this study. From the bottom to top of each box, the five numerical points represented the minimum observed value (lower edge), 25% quantile, median, 75% quantile, and maximum observed value (upper edge), respectively. The hollow circle at the upper part of each box showed the outlier

The incidence of IVH (stage 3 or 4) was lower in the OAC group than in the control group [2/127 (2%) vs. 9/125 (7%), RR 0.22 (95% CI 0.05, 0.99)]. In addition, the time to achieve full enteral feeding was shorter in the OAC group [23.13 ± 9.45 days vs. 28.50 ± 14.80 days] (Fig. 2). There were no statistical differences in the incidences of PDA, BPD, PVL, ROP, cholestasis or other secondary outcomes between the two groups.

### A comparison of the ELBW infants in the OAC and control groups is shown in Table 3

There was one case with stage 3A NEC in the control group, and this infant underwent surgery. In the OAC group, 1 case had stage 2A NEC that did not necessitate surgery. The incidence of NEC (Bell stage 2 or 3) among the ELBW infants in the OAC group was not different from that in the control group [(1/14 (7%) vs. 1/11 (9%), RR 0.79 (95% CI 0.06, 11.20)]. There were 4 cases of late-onset sepsis in the control group, including 1 case of clinical sepsis and 3 cases of proven sepsis (1 case of *Candida haemulonii*, 1 of *E. cloacae*, and 1 of *K. pneumoniae*), whereas there were no late-onset sepsis cases in the intervention group. The incidence of late-onset sepsis among the ELBW infants was lower in the OAC group than in the control group. The incidence of IVH (stage 3 or 4) and the time to achieve full enteral feeding for the ELBW infants were lower and shorter in the OAC group than in control group, respectively [1/14 (7%) vs. 6/11 (55%), RR 0.13 (95% CI 0.02, 0.93); 34.07 ± 8.15 days vs. 48.55 ± 22.00 days] (Table 2, Fig. 2). No statistical differences in other secondary outcomes were noted between the two groups.

**Table 3:** Clinical outcomes of extremely low birthweight infants between the OAC group and control group at the time of discharge

Clinical outcome	OAC group (*N* = 14)	Control group (*N* = 11)	*t/x*^*2*^-value	*P*-value	*Relative risk/Mean difference (95% CI)*	*Rate difference* *(95% CI)*
NEC (Bell stage 2 or 3), n (%)	1 (7.14)	1 (9.09)	-^a^	1.00	0.79(0.06, 11.20)	1.95%(− 28.01, 36.41%)
Bell stage 2, n (%)	1 (7.14)	0 (0.00)	-^a^	1.00	-^c^	7.14%(−25.71, 35.83%)
Bell stage 3, n (%)	0 (0.00)	1 (9.09)	-^a^	0.44	-^c^	9.09%(−19.02, 42.88%)
Late onset sepsis, n (%)	0 (0.00)	4 (36.36)	-^a^	0.03	-^c^	36.36%(0.42, 68.39%)
Clinical sepsis, n (%)	0 (0.00)	1 (9.09)	-^a^	0.44	-^c^	9.09%(−19.02,42.88%)
Proven sepsis, n (%)	0 (0.00)	3 (27.27)	-^a^	0.07	-^c^	27.27%(−6.1, 60.68%)
Patent ductus arteriosus, n (%)	6 (42.86)	8 (72.73)	-^a^	0.23	0.59(0.29, 1.19)	29.87%(−13.4, 61.11%)
Intraventricular hemorrhage (grade 3 or 4), n (%)	1 (7.14)	6 (54.54)	-^a^	0.02	0.13(0.02, 0.93)	47.40%(5.9, 75.55%)
Periventricular leukomalacia, n (%)	0 (0.00)	0 (0.00)	–	–	–	–
Bronchopulmonary dysplasia, n (%)	3 (21.43)	3 (27.27)	-^a^	1.00	0.79(0.20, 3.16)	5.84%(−29.98, 42.77%)
Retinopathy of prematurity, n (%)	7 (50.00)	4 (36.36)	-^a^	0.69	1.38(0.54, 3.52)	13.64%(−27.59, 48.99%)
Cholestasis, n (%)	1 (7.14)	3 (27.27)	-^a^	0.29	0.26(0.03, 2.18)	20.13%(−14.81, 54.22%)
Age of achieving full enteral feeding (d), mean ± SD	34.07 ± 8.15	48.55 ± 22.00	−2.28 (df23)	0.03	−14.47(−27.60, − 1.35)	-^b^
Age of achieving full oral feeding (d), mean ± SD	61.07 ± 15.13	62.91 ± 23.89	−0.24 (df23)	0.82	−1.84(− 18.03, 14.35)	-^b^
Rate of weight gain (kg.d), mean ± SD	17.75 ± 3.69	16.38 ± 3.11	0.99 (df23)	0.33	1.37(−1.51, 4.25)	-^b^
Indwelling time of PICC (d), mean ± SD	36.93 ± 11.53	40.54 ± 14.59	−0.69 (df23)	0.50	−3.62(−14.41, 7.18)	-^b^
Days of mechanical ventilation (d), mean ± SD	6.07 ± 6.49	14.64 ± 28.84	−1.08(df23)	0.29	−8.56(−24.93, 7.80)	-^b^
Days of noninvasive ventilation (d), mean ± SD	38.71 ± 17.88	32.00 ± 15.73	0.98 (df23)	0.34	6.71(−7.43, 20.86)	-^b^
Length of hospitalization, mean ± SD	71.00 ± 17.37	74.64 ± 20.60	−0.48 (df23)	0.64	−3.64(−19.34, 12.07)	-^b^

*IQR* interquartile range, *SD* standard deviation, *PICC* peripherally inserted central catheter

^a^ Fisher’s exact test

^b^ Measurement data: Rate difference and 95% CI cannot be calculated

^c^ Relative risk and 95% CI cannot be calculated

### A comparison of the sicker infants in the OAC group and control group is shown in Table 4

The incidence of NEC (Bell stage 2 or 3) among the sicker infants was lower in the OAC group than in the control group [1/20 (5%) vs. 9/22 (41%), RR 0.12 (95% CI 0.02, 0.88)]. The incidence of late-onset sepsis among the sicker infants in the OAC group was not different from that in the control group [3/20 (15%) vs. 9/22 (41%), RR 0.37 (95% CI 0.12, 1.17)]. The time to achieve full enteral feeding for the sicker infants was shorter in the OAC group than in the control group [30.40 ± 8.82 days vs. 44.68 ± 18.95 days]. No statistical differences in other secondary outcomes were noted between the two groups.

**Table 4:** Clinical outcomes of sicker infants between the OAC group and control group at the time of discharge

Clinical outcome	OAC group (*N* = 20)	Control group (*N* = 22)	*t/x*^*2*^-value	*P*-value	*Relative risk/Mean difference (95% CI)*	*Relative risk /Mean difference* (95% CI) *	*Rate difference* *(95% CI)*
NEC (Bell stage 2 or 3), n (%)	1 (5.00)	9 (40.91)	-^a^	**0.01**	0.12(0.02, 0.88)	0.12(0.02, 0.97)	35.91%(6.60, 58.82%)
Bell stage 2, n (%)	1 (5.00)	6 (27.27)	-^a^	0.10	0.18(0.02, 1.39)		22.27%(−4.69, 45.92%)
Bell stage 3, n (%)	0 (0.00)	3 (13.64)	-^a^	0.23	-^c^		13.64%(−8.79, 35.96%)
Late onset sepsis, n (%)	3 (15.00)	9 (40.91)	3.45	0.06	0.37(0.12, 1.17)		25.91%(−4.87, 50.89%)
Clinical sepsis, n (%)	2 (10.00)	3 (13.64)	-^a^	1.00	0.73(0.14, 3.95)		3.64%(−21.58, 27.44%)
Proven sepsis, n (%)	1 (5.00)	6 (27.27)	-^a^	0.10	0.18(0.02, 1.39)		22.27%(−4.69, 45.92%)
Patent ductus arteriosus, n (%)	11 (55.00)	11 (50.00)	0.11	0.75	1.10(0.62, 1.96)		5.00%(−26.24, 34.96%)
Intraventricular hemorrhage (grade 3 or 4), n (%)	1 (5.00)	4 (18.19)	-^a^	0.35	0.28(0.03, 2.26)		13.18%(−11.92, 36.49%)
Periventricular leukomalacia, n (%)	0 (0.00)	0 (0.00)	–	–	–		–
Bronchopulmonary dysplasia, n (%)	4 (20.00)	8 (36.36)	1.38	0.24	0.55(0.20, 1.55)		16.36%(−14.05, 42.81%)
Retinopathy of prematurity, n (%)	4 (20.00)	5 (22.73)	-^a^	1.00	0.88(0.27, 2.83)		2.73%(−25.31, 29.42%)
Cholestasis, n (%)	0 (0.00)	4 (18.19)	-^a^	0.11	-^c^		18.18%(−5.28, 41.01%)
Age of achieving full enteral feeding (d), Mean ± SD	30.40 ± 8.82	44.68 ± 18.95	−3.08(df40)	**0.01**	−14.29(−23.66, −4.91)	−8.29(− 16.31, −0.27)	-^b^
Age of achieving full oral feeding (d), Mean ± SD	52.65 ± 21.62	55.91 ± 20.27	−0.50(df40)	0.62	−3.26(− 16.32, 9.80)		-^b^
Rate of weight gain (kg.d), mean ± SD	16.28 ± 3.84	14.49 ± 3.32	1.63(df40)	0.11	1.80(−0.44, 4.03)		-^b^
Days of mechanical ventilation (d), mean ± SD	6.45 ± 4.86	11.00 ± 20.12	−0.99(df40)	0.33	−4.55(−13.89, 4.79)		-^b^
Days of noninvasive ventilation (d), mean ± SD	31.50 ± 18.89	28.50 ± 15.82	0.56(df40)	0.58	3.00(−7.83, 13.83)		-^b^
Indwelling time of PICC (d), mean ± SD	30.60 ± 15.99	30.68 ± 14.39	−0.02(df40)	0.99	−0.08(−9.55, 9.39)		-^b^
Length of hospitalization, mean ± SD	60.50 ± 23.02	62.27 ± 19.54	−0.27(df40)	0.79	−1.77(−15.05, 11.50)		-^b^

Sicker infants were defined as infants whose mechanical ventilation was started within the first 3 postnatal days and continued for at least 3 days or until death. SD: standard deviation. PICC: peripherally inserted central catheter

^a^ Fisher’s exact test

^b^ Measurement data: rate difference and 95% CI cannot be calculated

^c^ RR and 95% CI cannot be calculated

* The relative risk/mean difference was adjusted for birthweight (g), gestational age (weeks), and histologic chorioamnionitis

### A comparison of the histologically diagnosed maternal chorioamnionitis-exposed infants in the OAC group and control group

The 75 infants in this study included 33 in the control group and 42 in the OAC group. The incidences of NEC (Bell stage 2 or 3) and late-onset sepsis among the infants in the OAC group were not different from those in the control group [0/42 (0%) vs. 3/33 (9%), *P* = 0.08; 5/42 (12%) vs. 4/33 (12%), *P* = 1.00, RR 0.98 (95% CI 0.29, 3.37)], and the incidences of proven sepsis [3/42 (7%) vs. 4/33 (12%), *P* = 0.69, RR 0.59 (95% CI 0.14, 2.45)] and clinical sepsis [2/42 (5%) vs. 0/33 (0%), *P* = 0.50] were the same.

## Discussion

This prospective, pilot, single-center, 1:1 parallel RCT showed a potential lower incidence of NEC (Bell stage 2 or 3) and late-onset sepsis in preterm infants with GA ≤ 32 weeks who received an OAC intervention. In addition, the incidence of IVH (stage 3 or 4) was lower and the time to achieve full enteral feeding was shorter in the OAC group. In the subgroup analysis of the ELBW infants, some evidence indicated that the trend for the incidences of late-onset sepsis and IVH (stage 3 or 4) and the time to achieve full enteral feeding were lower and shorter, respectively. In the subgroup analysis among the sicker infants, there was some evidence that the trends in the incidence of NEC (Bell stage 2 or 3) and the time to achieve full enteral feeding were lower and shorter, respectively. No statistical differences were noted in the subgroup analysis among the histologically diagnosed maternal chorioamnionitis-exposed infants in the incidence of NEC and late-onset sepsis. No adverse reactions were observed in either group.

### OAC procedures

In the published literature, OAC could be applied for 5 d after birth or longer, even extending until the baby could receive partial oral feed or reach the correct GA at 32 weeks [25]. Colostrum is defined as breast milk produced within 5 days after delivery; it is rich in immunologic cellular activity factors and growth factors, the concentrations of which are high until 14–20 days after birth, especially in breast milk from mothers of premature infants [26, 27]. Therefore, in theory, an OAC duration that extends as long as possible will bring more benefits to premature infants. Colostrum from the infants’ own mothers was selected in our study, and the established 10-day OAC period provided these preterm infants more benefits without undue burden to the nurses.

Wide variations in the OAC procedure, including variation in the dosage of colostrum administered (ranging from 0.1 ml to 0.4 ml, even to 1.0 ml), the duration of each administration (only described “drop into the oral mucosa” or apply for less than 5 sec), the frequency of treatments for each day (every 2 to 6 h), the duration of the treatment protocol (ranging from 2 to 7 days, or on an as-needed basis), the type of applicator (syringe or swab), and the type of breast milk (fresh or frozen), have been reported in the published literature [25, 28–30]. In our study, a colostrum volume of 0.4 ml was the average volume used based on the previously published studies, and the 2-min duration of each administration was based on the experience of the nurses in our NICU. Moreover, our study used sterile silicone fingerstalls to smear the colostrum in the mouth, aiming to reduce the possible absorption of active substances in breast milk by cotton swabs. We also used disposable clean and sealed milk storage cups for breast milk collection to reduce secondary pollution during transfer.

### Safety of OAC

There were no adverse events, including oral mucosal damage, apnea, bradycardia, tachycardia or desaturation in our study, even in the ELBW infants and sicker infants who required mechanical ventilation starting within the first three postnatal days and continuing through at least 3 d. Therefore, OAC may be considered a safe procedure in the NICU for preterm infants.

### OAC for preventing NEC

NEC is a common complication in preterm infants that leads to many adverse consequences, which can increase the incidence of mortality and morbidity [31]. OAC is recognized as a new precaution against NEC [12, 32]. Biological studies have shown that OAC affects the prevention of NEC through the following mechanisms: (a) The cytokines in breast milk interact with the oropharyngeal lymphoid tissue system, which causes T-cell activation and leads to immune activation and anti-inflammatory reactions of various terminal organs. (b) Many protective biological factors in breast milk are absorbed by the oral mucosa and the gastrointestinal tract to play a role in promoting maturation, anti-inflammation and barrier protection. (c) Antioxidants in breast milk (such as peroxides and superoxide dismutase) can directly protect the immune cells in the oropharynx and can reach the intestinal tract to protect the mucosal barrier from oxidative damage, preventing oxidative stress-induced intestinal flora changes and translocation [33–36]. Although biological theory supports the results of these studies, most clinical studies and meta-analyses published in recent years have indicated that OAC cannot reduce the incidence of NEC [11, 28, 29, 37].

Our results showed that the incidence of NEC in the OAC group was lower for preterm infants with GA ≤ 32 weeks (from 10.4 to 2.4%), and there was some evidence of this result in sicker infants (from 40.9 to 5.0%). No surgery was required among the infants with NEC (stage 3) in the OAC group. These results are consistent with those of a previous study on the standard feeding protocol in preterm infants by McCallie and his team. That study enrolled 147 premature infants with BW ≤ 1500 g and indicated that the incidence of NEC was decreased among infants with minimal oral feeding early after birth (VLBW: from 18.1 to 3.1%, ELBW: from 35.5 to 7.7%) [24]. Similar to our study findings, a larger colostrum dosage, longer duration and higher frequency of OAC may be the reasons for the decrease in NEC. However, there was no difference in the incidence of NEC among the ELBW and histologically diagnosed maternal chorioamnionitis-exposed infants in the subgroup analysis. Despite some evidence of a difference in the incidence of NEC in the subgroup analysis among sicker infants, the limitation of the insufficient sample size in those subgroups must be considered. Thus, the conclusions need to be interpreted more rigorously. OAC may have a potential advantage for decreasing the incidence of NEC, but this is not certain.

### OAC for preventing late-onset sepsis

Late-onset sepsis is a multifactorial disease. Delayed feeding, artificial feeding and antibiotics lead to mucosal colonization of pathogenic bacteria, and the mucosal barrier of preterm infants is weak; thus, various invasive operations may cause colonized pathogenic bacteria to cross the immature epithelial barrier and then enter the blood, ultimately causing infection [38]. Mother’s milk, particularly colostrum, is rich in cytokines and other immune agents that provide bacteriostatic, bactericidal, antiviral, anti-inflammatory, and immunomodulatory protective agents against infection by *OAC* [32, 39]. Our study showed that the incidence of late-onset sepsis was potentially lower in the OAC group among preterm infants with GA ≤ 32 weeks (from 13.6 to 4.7%), and there was some evidence of this result in ELBW infants (from 36.4 to 0.0%). Furthermore, the late-onset sepsis reduction may be associated with the shorter time to achieve full enteral feeding in our study.

An RCT by Lee, including 48 premature infants with GA ≤ 28 weeks, showed that OAC significantly reduced the incidence of clinical sepsis [40]. In Elgawad’s study, OAC did not reduce the incidence of proven sepsis, whereas the count of intraoral *K. pneumoniae* in the OAC intervention group showed a significant decreasing trend [41]. With further analysis of the types of pathogenic bacteria detected in proven sepsis cases, we also found that the incidence of fungal infection in the OAC group showed a significant decreasing trend. This trend may be related to protective biological factors in breast milk, such as lactoferrin, which can reduce the occurrence of invasive fungal infections through the inhibition of fungal colonization and overgrowth [42]. However, considering the variety of colonizing bacteria and conditional pathogens in different NICUs, the inhibitory effect of OAC on different pathogens needs to be further studied.

### Role of OAC in preventing other complications

IVH is an important cause of brain injury in premature infants. It was observed that the incidence of severe IVH decreased significantly in the OAC group, which was considered to be related to a possible mechanism in which the infection rate decreased after the application of OAC. The incidence of infection-induced hypoxia/acidosis also decreased, which finally resulted in a brain-protective effect.

Many studies have shown that OAC may also have the advantages of shortening the time to achieve full enteral feeding and full oral feeding, reducing the incidence of feeding intolerance [11, 29, 39, 41], increasing the body weight at the correct gestational age of 36 weeks [43], reducing the length of hospital stay [29, 30, 37], and prolonging the days of breast milk feed [44]. In our study, it was also observed that the time to achieve full enteral feeding was shorter in the OAC group, which is of great significance for the management of premature infants. A possible mechanism might be that OAC stimulates oropharyngeal receptors, which improves the motility and secretory and absorptive abilities of the gastrointestinal tract.

### Limitations

The risk of bias in our study was assessed by using the table “The Cochrane Collaboration’s tool”. The results included selection bias (random sequence generation-low risk, allocation concealment-low risk), performance bias (blinding of participants and personnel-low risk), detection bias (bundling of outcome assessment-low risk), attrition bias (incomplete outcome data-low risk), reporting bias (selective reporting-low risk), and other bias (the monitoring statistician was the study statistician, early termination of the study).

We acknowledge the limitations of conducting a pilot single-center trial. The sample size was relatively small, especially with respect to the infants in each subgroup, so there may be deviations in the results. According to the sample size calculation results, the planned period of our research was 3 years, and the target recruitment population was 750 participants. After receiving three interim analysis reports after one and a half years of the trial, the sample size was recalculated as 260 participants based on the decline in NEC occurrence (from 10.0 to 2.0%). We terminated the study after 21 months for following reasons: 1) judging from the results of interim analysis, the effect of OAC was quite obvious; 2) the outbreak of COVID-19 in China led to a sharp decline in the number of premature deliveries and hospitalizations in our hospital, which would have greatly extended the study period; and 3) the frequency of parents transporting breastmilk to the hospital decreased significantly to avoid cross infection caused by personnel flow, which might weaken the effect of OAC and bias the outcomes. There are disadvantages based on the early termination of the study. For example, the incidence of false positive results is potentially increased. In addition, it is possible that the OAC was not effective or that the effect was not as great as the present results show. It is possible that the evidence is not sufficient to modify the current clinical practice. Considering the reasons above and possible biases, we suspended the study and published this research as a pilot study to obtain estimates for a sample size calculation for a larger, multicenter study.

In our study, the monitoring statistician conducting the interim analysis was the study statistician due to resource and personnel limitations. Although the statistician’s familiarity with the trial was conducive to interpreting the data, knowledge of the interim analysis results could potentially affect the management decisions of the trial, which could induce bias. The statistician in our study was not employed by the PI or funding sponsors. In addition, the DMC rigorously implemented the pre-established operating procedures to ensure the confidentiality of interim results, mitigate the possible bias and preserve the trial accuracy and integrity.

Biological research should also be performed to address the immune effects in recipient infants. There was a lack of assessment of long-term outcomes. Thus, large, multicenter, well-designed trials are required to more precisely and reliably evaluate the effects of OAC on short-term and long-term outcomes in preterm infants.

## Conclusions

This study strengthens the notion that OAC is a simple and feasible procedure without any additional risk to preterm infants with GA ≤ 32 weeks. There is a trend of positive efforts in decreasing the incidence of necrotizing enterocolitis, late-onset sepsis, and severe incidence of intraventricular hemorrhage, and this intervention may facilitate faster achievement of full enteral feeding. However, the potential advantages were less significant in ELBW and histologically diagnosed maternal chorioamnionitis-exposed infants. Future large, multicenter, well-designed studies are needed to confirm the effects of oropharyngeal administration of colostrum.

## Data Availability

The datasets during and/or analyzed during the current study are available from the corresponding author on reasonable request.

## Abbreviations

BW: Birthweight
BPD: Bronchopulmonary dysplasia
ELBW: Extremely low birthweight
GA: Gestational age
IVH: Intraventricular hemorrhage
NEC: Necrotizing enterocolitis
NICU: Neonatal intensive care unit
OAC: Oropharyngeal administration of colostrum
PDA: Patent ductus arteriosus
PVL: Periventricular leukomalacia
PASS: Power analysis and sample size
ROP: Retinopathy of prematurity
TRIPS: Transport Risk Index of Physiologic Stability
VLBW: Very low birthweight
